# Sericin/polyvinyl alcohol hydrogel optimization for enhanced angiogenesis: a promising strategy for treating chronic osteomyelitis

**DOI:** 10.1371/journal.pone.0328846

**Published:** 2025-07-24

**Authors:** Chayanee Noosak, Pavarish Jantorn, Suvimol Surassmo, Sittichat Chukaew, Jirut Meesane, Dennapa Saeloh Sotthibandhu

**Affiliations:** 1 Department of Medical Technology, School of Allied Health Sciences, Walailak University, Nakhon Si Thammarat, Thailand; 2 Faculty of Medical Technology, Prince of Songkla University, Songkhla, Thailand; 3 National Nanotechnology Center (NANOTEC), National Science and Technology Development Agency (NSTDA), Pathum Thani, Thailand; 4 Department of Mining and Materials Engineering, Faculty of Engineering, Prince of Songkla University, Songkhla, Thailand; 5 Department of Biomedicine and Biomedical Engineering, Faculty of Medicine, Prince of Songkla University, Songkhla, Thailand; University of Waterloo, CANADA

## Abstract

Chronic osteomyelitis, often accompanied by bone loss, requires an adequate angiogenic response for bone regeneration. Loading growth factors into a drug vehicle to promote angiogenesis can address this challenge. In a previous study, we demonstrated the potential of sericin/polyvinyl alcohol (PVA) hydrogel as a functional biomaterial carrier for osteomyelitis treatment. In this study, we optimized sericin/PVA hydrogel for enhanced angiogenesis by supplementing sericin nanoparticles as vascular endothelial growth factor (VEGF) nanocarriers. Sericin nanoparticles, 284.20 ± 13.20 nm in size, exhibited a spherical morphology with 86% VEGF encapsulation efficiency. After integrating VEGF-loaded sericin nanoparticles, the hydrogel was coated with 0.1% and 1% gelatin, and its physical and mechanical properties were assessed. Coating the hydrogel with gelatin enhanced its swelling properties, providing an appropriate degradation rate to support bone regeneration and angiogenesis, and improve mechanical properties. The uncoated hydrogel and hydrogels coated with 0.1% and 1% gelatin exhibited burst release rates of 70%, 60%, and 45% with cumulative release rates on day 14 measured at 76%, 67%, and 57%, respectively. The hydrogels were biocompatible with MC3T3-E1 osteoblastic cell lines and human umbilical vein endothelial cells (HUVEC). The gelatin-coated hydrogels also promoted cell attachment of HUVEC cells. Gelatin-coated hydrogels containing VEGF-loaded sericin nanoparticles were evaluated for their bioactivity on HUVEC cell proliferation. After a 14-day treatment, cell proliferation in 0.1% gelatin-coated hydrogel was significantly higher than in 1% gelatin-coated hydrogel, with over a 160% increase. The expression levels of genes related to angiogenesis were quantitatively examined and results suggested that the hydrogels affected the eNOS pathway to promote angiogenesis. Despite optimization efforts, the sericin/PVA hydrogel maintained effective antibacterial activity against Gram-positive and Gram-negative bacteria. The enhanced sericin/PVA hydrogel showed promise as a novel implant biomaterial for treating chronic osteomyelitis, particularly by promoting angiogenesis.

## Introduction

Osteomyelitis is an infection of the bone, usually caused by bacteria, which generate a build-up of pus as an abscess within the bone, limiting the blood supply. The symptoms of chronic osteomyelitis include pain and disability. Treatment requires adequate surgical debridement, often leading to the loss of large soft tissue and bone. Ineffective treatment can result in death [1].

Antibiotic-loaded poly (methyl methacrylate) (PMMA) bone cement is the gold standard for local chronic osteomyelitis treatment. However, on the downside, PMMA does not degrade naturally, with additional surgery needed for removal [2]. This repetition of surgical procedures increases the risk of wound infection and escalates treatment costs. PMMA bone cement cannot act as a scaffold for bone regeneration [3]. To address these limitations, the use of local antibiotic from a biodegradable carrier with biological functions is an attractive option.

Organic-inorganic hybrids or composite biomaterials are now increasingly used to improve traditional drug delivery systems [4–6]. Previously, hybrid hydrogels of polyvinyl alcohol (PVA)-based antibiotic carriers were used as cartilage scaffolds for osteoarthritis surgery [7]. PVA is suitable as a scaffold for bone tissue engineering because of its physical stability and can be further modified by adding bioactive molecules to enhance bone tissue formation [8]. Sericin is a natural silk protein polymer produced by silkworms. Gummy sericin, removed during raw silk production, is an underutilized by-product from the silk textile industry. This silk protein is particularly attractive because of its biocompatibility and biodegradation. Sericin has diverse bioactivities such as anti-apoptosis, antioxidant, anti-inflammation, non-immunogenicity, and cell proliferation [9–12]. The surface coating of biomaterials can control drug release, improve mechanical properties, and influence cellular behavior by promoting cell attachment to the scaffold. Gelatin, a natural polymer derived from collagen, contains Arg-Gly-Asp (RGD) sequences, which enhance cell adhesion by binding to integrin proteins on the cell membrane [13]. The biocompatibility of gelatin and its ability to mimic the microstructure of the extracellular matrix make it an ideal candidate for biomaterial coating and surface modification [14]. Recent studies demonstrated that gelatin coating improved cell adhesion and proliferation on various scaffold types including polycaprolactone, β-tricalcium phosphate, and electrospun polycaprolactone/graphene oxide [15–17].

In a previous study, we first designed and then observed the function of sericin/PVA hydrogel incorporated with ciprofloxacin for treating bone infection [18]. Ciprofloxacin-loaded sericin/PVA hydrogel was stable and showed enhanced mechanical strength after freeze-thawing without the need for chemical additives. Sericin/PVA hydrogel at a volume ratio of 75:25 showed good swelling property, degradation rate, and drug release. The hydrogel also successfully released ciprofloxacin to inhibit bacteria and enhance the proliferation of osteoblastic cell lines.

Most cases of chronic osteomyelitis involve bone loss, rendering the enhancement of osteogenesis as the goal of the treatment. Angiogenesis also plays an important role in bone regeneration, while the deficiency of nutrients and oxygen causes non-uniform cell differentiation or cell death [19]. Loading of growth factor in a drug vehicle to promote angiogenesis can address this issue. Vascular endothelial growth factor (VEGF) is regarded as the most effective growth factor in angiogenesis, with the primary ability to induce neovascularization [20]. Proteins are crucial for endothelial cell proliferation, migration, and activation. VEGF serves as a significant indicator of vascularization in bone tissue and influences bone growth in response to biomaterials through gene expression and protein levels [21]. Sericin-based biomaterials incorporated with VEGF have been successfully used in wound management applications [22]. However, the impact of utilizing a sericin scaffold containing VEGF to coordinate osteogenesis and angiogenesis has not been previously examined.

**Fig 1:**
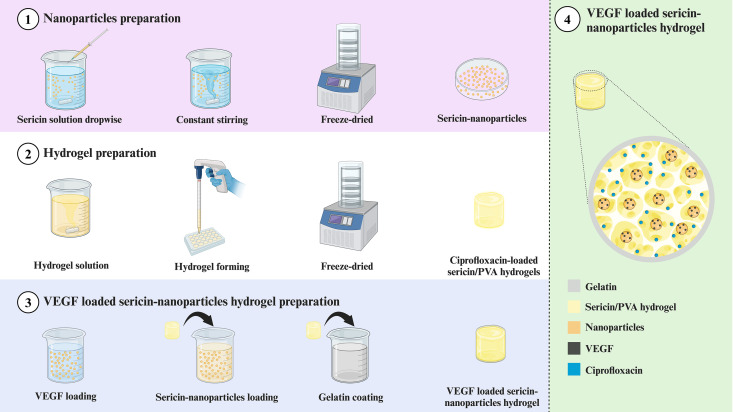
Schematic illustration of sericin/PVA hydrogel containing VEGF-loaded nanoparticles.

This study optimized sericin/PVA hydrogel for angiogenesis promotion in chronic osteomyelitis treatment. VEGF-loaded sericin nanoparticles were prepared and loaded into sericin/PVA hydrogel (Fig 1). The hydrogel was coated with gelatin to control the delivery of VEGF and an antibiotic. The physical properties of the sericin/PVA hydrogel containing VEGF-loaded nanoparticles were characterized, and the biological properties of the hydrogels including cell compatibility, and angiogenic and antibacterial activities were investigated. The gene-related angiogenic expression was also observed. Sericin/PVA hydrogel containing VEGF-loaded nanoparticles was hypothesized as a promising biomaterial implant for chronic osteomyelitis treatment to facilitate angiogenesis.

## Materials and methods

### Nanoparticle preparation and characterization

#### Fabrication of sericin nanoparticles.

Sericin was extracted according to our previous study [23], using the desolvation technique to prepare sericin nanoparticles, with poloxamer 407 as a desolvating agent. Briefly, 10 mL of sericin solution (1% w/v) was added dropwise to 10 mL of poloxamer 407 at room temperature with constant stirring. The visible sericin nanoaggregates were then centrifuged at 23500 g for 10 min to collect the precipitates. The sample was purified with repeated centrifugation at 13400 g for 10 min in deionized water. To remove poloxamer 407 from the sericin nanoparticle suspension, the pellet was sonicated in deionized water at 40% amplitude for 15 min, and the sericin nanoparticles were lyophilized for further use.

#### Sericin nanoparticle characterization.

Dynamic light scattering (DLS; 3000, HS, UK) was used to evaluate the polydispersity index (PDI), size, and surface charge of the sericin nanoparticles. To obtain uniform dispersion, the lyophilized sericin nanoparticles were suspended in filtered deionized water and then sonicated at 50% amplitude for 20 min. The DLS measurement was repeated three times at 25°C with a fixed detection angle of 90°.

The morphology of the sericin nanoparticles was investigated by scanning electron microscopy (SEM) analysis (JEOL, Japan). The sericin nanoparticle solution was mounted onto stubs and coated with gold before SEM observation.

#### VEGF encapsulation.

To prepare VEGF-loaded sericin nanoparticles, 20 μL of milli-Q water containing 50 ng of VEGF (Sigma-Aldrich, USA) was mixed with 1 mL of sericin nanoparticle solution (5 mg/mL). The mixture was incubated at 4°C for 16 h and then centrifuged at 10000 rpm for 10 min. The supernatant was collected and the VEGF encapsulation efficiency (EE) was calculated as:


$$\%\text{EE}=\frac{\text{total\textbackslash{} amount\textbackslash{} of\textbackslash{} drug\textbackslash{} - \textbackslash{} amount\textbackslash{} of\textbackslash{} unbound\textbackslash{} drug}}{\text{total\textbackslash{} amount\textbackslash{} of\textbackslash{} drug}}\text{x}100$$

#### Hydrogel preparation.

Ciprofloxacin-loaded sericin/PVA hydrogels were prepared by the freeze-thaw method without using chemical agents, following a previous study [18]. Briefly, 2% (w/v) of sericin solution and 10% (w/v) of PVA solution at a volume ratio of 75:25 were mixed and stirred continuously. Ciprofloxacin solution at a final concentration of 5 mg/mL was added, and the mixture solution was frozen and thawed for 3 cycles. To prepare sericin/PVA hydrogels containing VEGF-loaded sericin nanoparticles, the hydrogels were immersed in 300 μl of VEGF-loaded sericin nanoparticles at 4°C for 5 h and then freeze-dried. Gelatin solutions at 0.1 and 1% (w/v) were used for coating the hydrogel to prepare gelatin-coated sericin/PVA hydrogels containing VEGF-loaded sericin nanoparticles. The gelatin solution was prepared by dissolving gelation powder in deionized water at 50°C for 1 h with stirring. The sericin/PVA hydrogels were then immersed in a gelatin solution containing 0.1% glutaraldehyde at room temperature for 30 min with continuous stirring. The coated hydrogels were freeze-dried and stored at room temperature until use.

### Hydrogel characterization

#### Swelling property.

The swelling ratio of the hydrogels was determined by immersion in phosphate-buffered saline (PBS) at 37°C for 5, 10, 20, 30, 60, 90, 120, 150, 180, and 360 min. After removal of excess PBS, the hydrogels were immediately weighed. The swelling ratio was calculated using the equation:


$$\text{Swelling\textbackslash{} ratio}=\frac{\text{weight\textbackslash{} of\textbackslash{} \textbackslash{} swollen\textbackslash{} hydrogel\textbackslash{} - weight\textbackslash{} of\textbackslash{} dry\textbackslash{} hydrogel}}{\text{weight\textbackslash{} of\textbackslash{} \textbackslash{} dry\textbackslash{} hydrogel}}$$

#### Enzymatic degradation.

The freeze-dried hydrogels were weighed and then immersed in lysozyme solution (2 mg/mL) at 37°C for 1, 3, 5, 7, and 14 days. At specific time points, the hydrogels were rinsed with distilled water and freeze-dried using a freeze dryer. The degradation of the hydrogels was calculated by measuring the weight loss according to the equation:


$$\;\%\text{Weight\textbackslash{} loss}=\frac{\text{initial\textbackslash{} weight\textbackslash{} - \textbackslash{} weight\textbackslash{} after\textbackslash{} degradation}}{\text{initial\textbackslash{} weight}}\text{x}100$$

#### Differential scanning calorimetry (DSC) analysis.

The thermal behavior of the hydrogels was assessed by a Differential Scanning Calorimeter (DSC3 + , Mettler Toledo, Switzerland). The hydrogels were weighed, placed in aluminum pans, and covered. The pans were then subjected to controlled heating from 25°C to 300°C at a rate of 10°C/min in the DSC instrument under a nitrogen atmosphere.

#### Compressive strength evaluation.

The compressive strength of the hydrogels was evaluated by a Universal Testing Machine (Lloyd Instruments, UK). Each hydrogel sample was shaped into a cylinder with a diameter of 8 mm and a height of 7.5 mm before immersion in PBS at room temperature for 24 h. The testing was conducted at room temperature with a load of 10 N, applied at a rate of 1 mm/min, and stopped at a strain of 40%.

#### Evaluation of hydrogel release property.

The release of ciprofloxacin from the hydrogels was determined in phosphate buffer pH 7.4 at 37°C. At given time points, the release medium was collected and replaced with an equivalent volume of fresh phosphate buffer. The cumulative drug release was determined using UV spectrophotometry at 309 nm.

The drug release kinetics of the hydrogels were analyzed by data fitting using the Higuchi, Korsmeyer-Peppas, and zero-order kinetic models [24–26]. The data were analyzed by Python version 3.10, with the best-fit model identified by the coefficient of determination (R^2^).

### Assessment of hydrogel biological activity

#### Cell culture.

MC3T3-E1 cells were cultured in α-modified minimal essential medium (α-MEM, Gibco, USA) supplemented with 10% fetal bovine serum (FBS, Gibco, USA) and 1% antibiotic-antimycotic (Gibco, USA) at 37°C under 5% CO_2_ atmosphere.

HUVEC cells (ATCC, USA) were cultured using Kaighn’s Modification of Ham’s F-12 Medium (F-12K, ATCC, USA) supplemented with 10% FBS, 1% penicillin (Gibco, USA), 0.1 mg/mL of heparin solution (Sigma-Aldrich, USA), and 0.03 mg/mL of endothelial cell growth supplement (Sigma-Aldrich, USA) at 37°C under 5% CO_2_ atmosphere.

#### Biocompatibility testing.

The hydrogels were immersed in the culture medium at 37°C for 24 h, and the supernatant was collected for testing. The biocompatibility of the hydrogels was assessed by a LIVE/DEAD viability/cytotoxicity Kit (Thermo Fisher Scientific, USA) according to the manufacturer’s protocol. MC3T3-E1 and HUVEC cells were incubated with the hydrogel extract at 37°C under 5% CO_2_ atmosphere for 14 days. The hydrogels were then removed, and cells were washed with PBS and stained with 2 mM calcein AM and 4 mM ethidium homodimer-1 for 45 min at room temperature. The stained cells were visualized under a fluorescent microscope (ZEISS, Germany), with excitation and emission of green (ex/em 494/517 nm for Calcein AM) and red (ex/em 528/617 nm for EthD‐1) fluorescence.

#### Cell viability assay.

The viability of HUVEC cells on the hydrogels was determined using the MTT assay. HUVEC cells were seeded at a density of 1 × 10^4^ cells per well in a 96-well plate and incubated at 37°C under 5% CO_2_ atmosphere. After 24 h, the culture medium was removed, and 200 µL of the hydrogel extract was added to each well and cultivated at 37°C under 5% CO_2_ atmosphere for 7 and 14 days. The hydrogel extract was replaced every 3–4 days. At specific time points, the cell culture medium was removed. Then, 2 mg/mL of MTT solution was added to each well following incubation at 37°C under 5% CO_2_ for 4 h. After removal of the MTT solution, dimethyl sulfoxide was added to dissolve the formazan crystals. The absorbance was measured at 570 nm using a microplate reader.

#### Cell attachment visualization.

HUVEC cells were seeded on the hydrogels at a density of 1 × 10^5^ cells in 20 µL of growth medium for 2 h. The growth medium was then added to each well and cultured at 37°C under 5% CO_2_ atmosphere. After 7 days, the hydrogels were fixed with 4% paraformaldehyde, washed with distilled water, freeze-dried, coated with gold, and observed by SEM.

#### Investigation of angiogenesis-related genes.

To determine the expression of angiogenesis-related genes, HUVEC cells were seeded on the hydrogels and cultured at 37°C under 5% CO_2_ for 14 days. Total RNA of the HUVEC cells was isolated using a Total RNA Mini Kit (Geneaid Biotech Ltd, Taiwan). RNA purities and concentrations were measured by a nanodrop spectrophotometer. For single-strand complementary DNA (cDNA) synthesis, the purified RNA was reverse transcribed into cDNA using a SensiFAST™ cDNA Synthesis Kit (Bioline, UK), according to the manufacturer’s instructions.

The expression of the angiogenesis-related genes was evaluated by real-time quantitative polymerase chain reaction (RT-qPCR) assay. The levels of angiogenesis-related gene expressions including endothelial nitric oxide synthase (*eNOS*), hypoxia-inducible factor-alpha (*HIF-α*), stromal cell-derived factor-1 (*SDF-1*), vascular endothelial growth factor receptor 2 (VEGF-R2; *KDR*), and vascular endothelial growth factor A (*VEGF-A*) were quantified. The reactions were conducted using HOT FIREPol^®^ EvaGreen^®^ qPCR Mix Plus (no ROX) (Solis BioDyne, Estonia), with a CFX Connect real-time PCR System (Bio-Rad, USA) for real-time analysis. The Cq data were obtained using CFX Maestro software (Bio-Rad, USA), with the delta-delta Cq method utilized to calculate the relative gene expression. The housekeeping gene, glyceraldehyde-3-phosphate dehydrogenase (GAPDH), was used to normalize the expression levels. The targeted gene primers are listed in Table 1.

**Table 1:** Primer sequences for RT-qPCR of the related gene expressions.

Related genes	Sequence (5′ to 3′)	References
***eNOS***	Forward: ATCCAGTGGGGGAAGCTGCA	[27]
	Reverse: GGGAACACTGTGATGGCCGAG	
***HIF1-α***	Forward: GCCCTAACGTGTTATCTGTCGCT	
	Reverse: TGATTGCCCCAGCAGTCTACATG	
***SDF1-α***	Forward: CGTCAGCCTGAGCTACAGATGC	
	Reverse: TTGTTGTTCTTCAGCCGGGCT	
***KDR***	Forward: AGCCAGCTCTGGATTTGTGGA	[28]
	Reverse: CATGCCCTTAGCCACTTGGAA	
***VEGF-A***	Forward: CATCCAATCGAGACCCTGGTG	
	Reverse: TTGGTGAGGTTTGATCCGCATA	
***GAPDH***	Forward: TCGGAGTCAACGGATTTGGT	[29]
	Reverse: TTCCCGTTCTCAGCCTTGAC	

### Antibacterial activity testing

The zone of inhibition test was performed to evaluate the antibacterial activity of the hydrogels. *Staphylococcus aureus* ATCC 25923, *S. epidermidis* ATCC 12228, and *Escherichia coli* ATCC 25922 were overnight cultured in tryptic soy broth (TSB) at 37°C. The overnight cultures were prepared into the exponential phase of growth at 37°C for 3–4 h. The bacteria were adjusted to 0.5 McFarland turbidity standard and swabbed on Mueller Hinton agar (MHA) plates. The ciprofloxacin standard disk and ciprofloxacin released from the hydrogel absorbed into the paper disk were placed on the surface of the MHA plate. The inhibition zones of each plate were observed after incubation at 37°C for 16–18 h.

### Statistical analysis

All the experiments were performed in triplicate, with statistically significant differences between groups analyzed by one-way analysis of variance. The significance level was set at *p* < 0.05.

## Results and discussion

### Characterization of sericin nanoparticles

**Fig 2:**
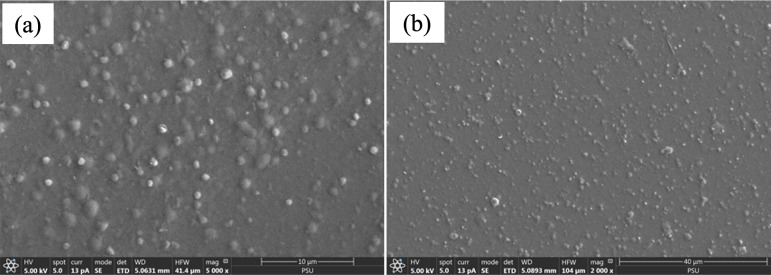
SEM image of sericin nanoparticles. (a) 5000X and (b) 2000X magnification.

Protein-based nanoparticles have been extensively developed for drugs or bioactive molecule delivery because they are easily biodegradable, which allows for efficient elimination from the body and reduces the risk of adverse effects [30]. Nanoparticles have shown the potential to improve shelf-life and control the release of encapsulated bioactive molecules. Sericin has been widely developed as a nanocarrier for drugs and bioactive molecules due to its biocompatibility and biodegradability [31,32]. Several synthesis techniques have been utilized to synthesize sericin nanoparticles [33]. In this study, sericin nanoparticles were synthesized by the desolvation technique for sustained release of VEGF. The nanoparticles were evaluated by size and surface (Table 2). The average particle size of sericin nanoparticles was 284.20 ± 13.20 nm. The surface charge, as indicated by the zeta potential, is an important factor for evaluating particle stability, particularly for nanoparticles [34]. In our study, the zeta potential of the sericin nanoparticles was −22.23 ± 0.49 mV, indicating high stability. The negative charges of nanoparticles were not toxic to the cell membrane [35]. SEM images revealed the morphology of sericin nanoparticles (Fig 2) as spherical and without obvious aggregation, confirming their stability for long-term sustained release.

**Table 2:** Particle size and zeta potential of sericin nanoparticles.

	Average	SD
**Particle size**	284.20	13.20
**Zeta potential**	−22.23	0.49

VEGF is crucial for signaling endothelial progenitor cells and stimulating endothelial cell proliferation to achieve angiogenesis, which is essential for bone regeneration in osteomyelitis patients [36]. However, the short half-life of VEGF limits its function in bone regeneration with blood vessels, and effective strategies for the controlled delivery of VEGF are needed. Encapsulating growth factors within nanoparticles is now widely used to prolong sustained release and maintain bioactivity. In this study, sericin nanoparticles were used as VEGF nanocarriers. The encapsulation efficacy (%EE) was measured to evaluate the VEGF concentration successfully loaded into the nanoparticles. Results revealed high values of 86% for VEGF-loaded sericin nanoparticle encapsulation efficiency. A previous study showed that VEGF encapsulated in nanoparticles provided sustained VEGF release, with a greater effect than free VEGF [37]. Another study demonstrated that using VEGF-loaded nanoparticles for long-term release enhanced neovascularization *in vivo* [38]. These results indicated that using sericin nanoparticles as VEGF nanocarriers was a promising strategy for high encapsulation efficiency and sustained VEGF release.

### Physical and mechanical properties of gelatin-coated hydrogel containing VEGF-loaded sericin nanoparticles

**Fig 3:**
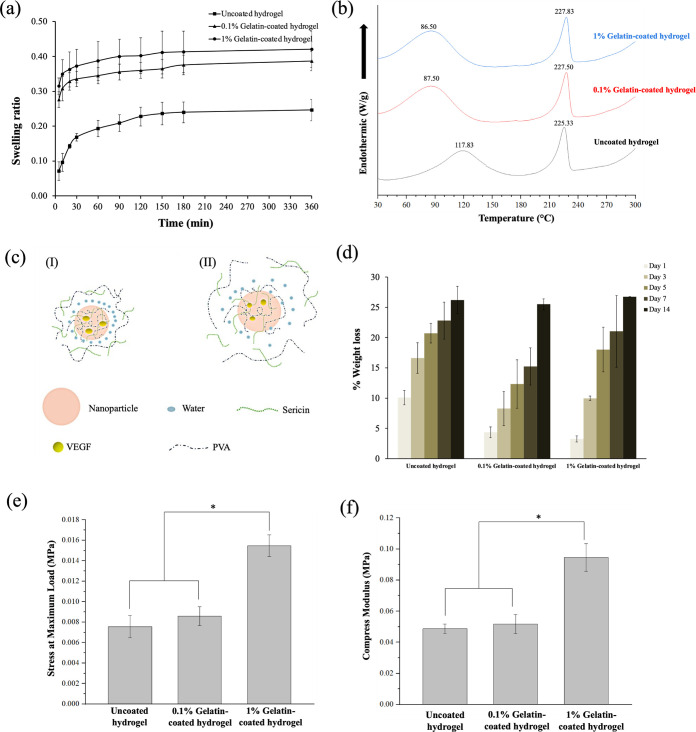
Physical and mechanical properties of gelatin-coated hydrogels containing VEGF-loaded sericin nanoparticles. (a) Swelling ratio at different times, (b) DSC thermograms, (c) organization of VEGF-loaded nanoparticles at the interfacial area of the hydrogel matrix via bound water: (I) uncoated hydrogel and (II) coated hydrogels, (d) degradation rate of the hydrogels in lysozyme solution, (e) stress at maximus, and (f) compressive strength. * represents a statistically significant difference (**p* *< 0.05).

Swelling behavior is a key hydrogel property parameter that facilitates nutrient transport to support cell attachment and proliferation. The swelling of all hydrogels was measured up to 6 h with the swelling ratio as a function of time, as shown in Fig 3a. All the hydrogels revealed high swelling ratios within 30 min and thereafter a slight increase to reach equilibrium after 3 h. The 0.1% and 1% gelatin-coated hydrogels demonstrated a higher swelling ratio than the uncoated hydrogel. This correlated with the hydrophilic nature of gelatin, which can form hydrogen bonds with water molecules [39], leading to characteristic water absorption and swelling and resulting in higher swelling ratios of the gelatin-coated hydrogels. A previous study reported that the swelling ratio of the hydrogel increased with increasing gelatin concentration, indicating that the presence of gelatin promoted water absorption [40]. Therefore, coating with gelatin increased the swelling property of the hydrogel.

The DSC thermograms revealed peaks at 87.50°C and 86.5°C for the gelatin-coated hydrogels, representing hydration of the 0.1% and 1% gelatin-coated hydrogels, respectively. The uncoated hydrogel showed a peak at 117.83°C (Fig 3b). This demonstrated that the uncoated hydrogel had more stability and a more regular structure of water organization than the coated hydrogel. The uncoated hydrogel had more stable and regularly bound water molecules around the nanoparticles at the interfacial area of the hydrogel matrix. On the other hand, the coated hydrogel exhibited a non-regular structure of bound water molecules with more free space, facilitating nutrient transfusion. This result supported the enhancement of cell proliferation [41,42]. The proposed mechanism of organization is shown in Fig 3c. The melting peaks of PVA as the main component of the hydrogel were found at 225.33, 227.50, and 227.83°C for the uncoated, 0.1%, and 1% gelatin-coated hydrogel, respectively. Results demonstrated that the addition of gelatin enhanced the PVA melting temperature because the PVA molecules formed chemical grafting and crosslinking with gelatin via glutaraldehyde bonding [43].

Biodegradable scaffolds have been extensively studied for local application in chronic osteomyelitis to avoid postoperative surgery and provide space for tissue growth and matrix deposition [44], and biodegradable materials show promise as effective local delivery carriers. In this study, biodegradable polymers including sericin, PVA, and gelatin were used as biomaterial-based hydrogels due to their biodegradability. The degradation rate of the hydrogel was evaluated by soaking in lysozyme solution for 14 days, as shown in Fig 3d. All the hydrogels exhibited weight loss of around 25% within 14 days, with no significant difference in degradation rate between the 0.1% and 1% gelatin-coated hydrogels. The uncoated hydrogel demonstrated higher degradation rates at earlier time points than the gelatin-coated hydrogels because the gelatin layer served as a protective barrier, resulting in slow degradation in the early period. A rapid degradation rate may lead to treatment failure [45], and the rate of degradation must be controlled to support bone regeneration and angiogenesis. In our previous study [18], we determined that the gelatin-coated hydrogel reduced the degradation rate after 14 days. These results indicated that hydrogel coating was an effective approach to control the degradation rate and promote osteogenesis and angiogenesis in osteomyelitis.

Mechanical characteristics, commonly divided into cortical and cancellous classifications, are crucial for the construction of bone scaffolding [46]. The mechanical properties of the uncoated hydrogel and gelatin-coated hydrogels were assessed through compression tests (Fig 3e–f). The compression moduli for the 0.1%, 1% gelatin-coated, and the uncoated hydrogels were 0.052, 0.095, and 0.049 MPa, respectively. These results highlighted the significant enhancement in strength achieved by gelatin coating on the sericin/PVA hydrogel. Adding gelatin to the structure improved its mechanical properties [47].

### Drug release profile of the uncoated hydrogel and gelatin-coated hydrogels

**Fig 4:**
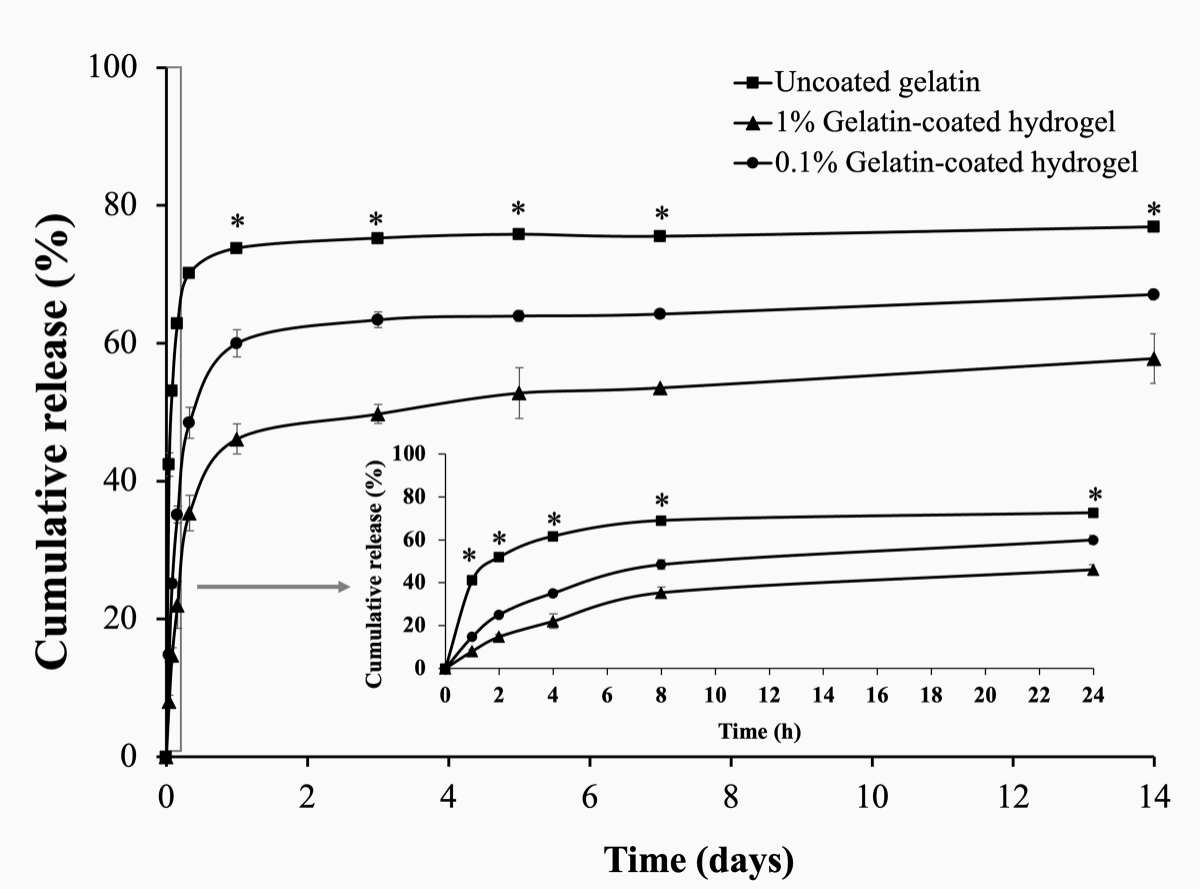
Drug release profile of the uncoated hydrogel and gelatin-coated hydrogels. * represents a statistically significant difference (*p *< 0.05).

The cumulative drug release was calculated to investigate the potential of hydrogel as a drug delivery carrier. A sustained drug release profile is essential to maintain a therapeutic level for the purpose of local antibiotic treatment. As shown in Fig 4, the *in vitro* cumulative release of ciprofloxacin in all hydrogel formulations demonstrated an initial burst release within the first 24 h of incubation, followed by a sustained release over 14 days. The initial burst release is necessary to prevent bacterial infection after surgery, followed by a prolonged release to maintain therapeutic concentration [48]. The hydrogel without gelatin coating had a high burst release of 70%, which decreased to 60% and 45% for the 0.1% and 1% gelatin-coated hydrogels, respectively. The cumulative release rates of the hydrogels measured on day 14 were 76%, 67%, and 57% for the uncoated hydrogel, 0.1%, and 1% gelatin-coated hydrogels, respectively. A significant cumulative release of ciprofloxacin was observed at all time points compared to the uncoated hydrogel. Coating hydrogel with gelatin effectively prolonged the release of ciprofloxacin. Gelatin coating biomaterial has been studied for controlled antibiotic release in bone tissue engineering. A gelatin-coated scaffold reduced burst release values as an effective initial antibacterial measure and promoted long-term protection against infection [49]. A previous report investigated a scaffold coated with gelatin to sustain the release of bioactive molecules in bone tissue engineering [50], while another study demonstrated a unique sustained release profile of gelatin-coated scaffolds, showing promising results in supporting homogeneous bone tissue formation [51]. These results suggested that gelatin coating can be used to control the release of drugs and bioactive molecules from hydrogels and improve their efficacy in local delivery systems.

The drug release behavior of hydrogels is influenced by several factors such as release mechanisms, environmental conditions, and physical properties [52]. In this study, the release mechanism of ciprofloxacin from the hydrogels was considered after the burst release phase by applying three different kinetic models as Higuchi, Korsmeyer-Peppas, and zero-order. As shown in Table 3, the R^2^ values for the Higuchi model ranged from 0.8849 to 0.9856, while the R^2^ values for the zero-order kinetic equation ranged from 0.8035 to 0.9071. The best-fitting model for all hydrogel conditions was the Korsmeyer-Peppas model, with the highest R^2^ of 0.9233 to 0.9928, indicating that the release of ciprofloxacin from the hydrogel was influenced by diffusion. The Korsmeyer-Peppas model describes the drug release mechanism through the release exponent (n) [25]. Release behavior can be classified into Fickian diffusion (n ≤ 0.45), non-Fickian diffusion (0.45 < n < 0.89), or case-II transport (n > 0.89) [53]. The exponent n values of the uncoated hydrogel, 0.1% gelatin-coated hydrogel, and 1% gelatin-coated hydrogel were 0.01, 0.04, and 0.08, respectively. These findings indicated that Fickian diffusion was the predominant mechanism for the diffusion of drug molecules from the hydrogel matrix.

**Table 3:** The constant (k), coefficient of determination (R^2^), and release exponent (n) of the Higuchi, Korsmeyer-Peppas and zero-order models.

Hydrogel conditions	Higuchi		Korsmeyer-Peppas		Zero order	
	k_h_ (mg/mL.h^1/2^)	R^2^	n	R^2^	k_0_ (mg/mL.h)	R^2^
**Uncoated hydrogel**	0.0102	0.8849	0.01	0.9233	0.0001	0.8035
**0.1% gelatin-coated hydrogel**	0.0230	0.9257	0.04	0.9413	0.0002	0.8483
**1% gelatin-coated hydrogel**	0.0387	0.9856	0.08	0.9928	0.0003	0.9071

### Biocompatibility of gelatin-coated hydrogels containing VEGF-loaded sericin nanoparticles with osteoblasts and HUVEC cells

**Fig 5:**
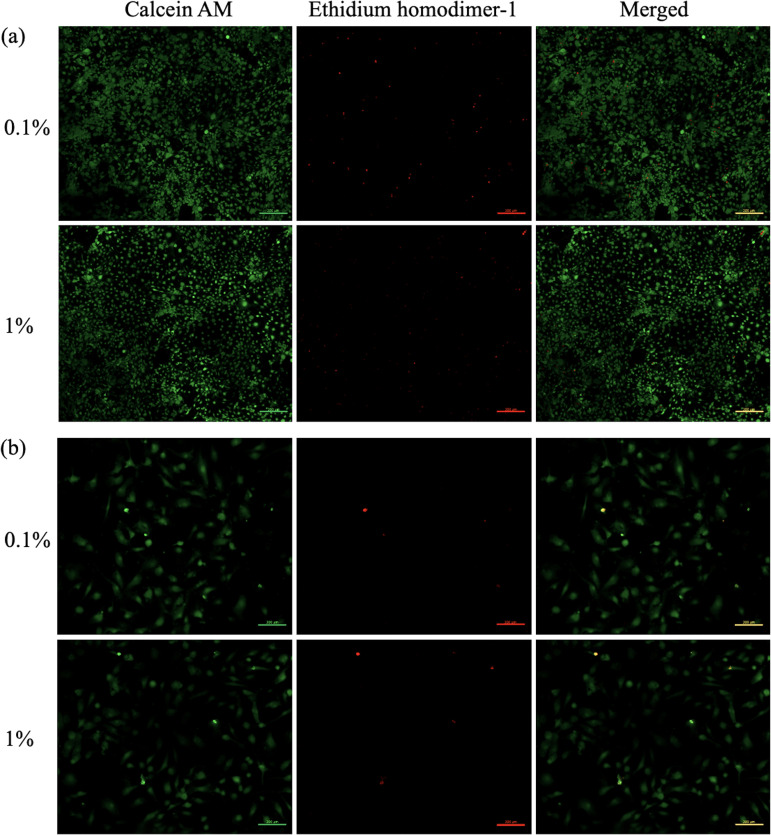
Biocompatibility of gelatin-coated hydrogels containing VEGF-loaded sericin nanoparticles on cell culture after calcein AM and ethidium homodimer-1 staining. Green color indicates living cells and red color indicates dead cells. MC3T3-E1 cells. (b) HUVEC cells.

A previous study reported that sericin/PVA hydrogel significantly stimulated the growth of osteoblast cells, suggesting its potential as an alternative option for treating chronic osteomyelitis [18]. In this study, the sericin/PVA hydrogel was modified by adding VEGF-loaded sericin nanoparticles and coating them with gelatin to promote angiogenesis, which is important for bone cell growth. Designing biomaterial scaffolds requires careful attention to biocompatibility. The gelatin-coated hydrogel was assessed for biocompatibility to osteoblast and HUVEC cells by a live/dead viability assay. After 14 days of treatment, most osteoblast and HUVEC cells were alive with green fluorescence in all hydrogels, indicating good biocompatibility. Some dead cells were present as evidenced by the red fluorescence (Fig 5). Thus, applying gelatin coating to the hydrogel did not detrimentally impact the osteoblasts or HUVEC cells. The hydrogel demonstrated excellent biocompatibility with both cell types, indicating its potential as a scaffold for facilitating bone tissue regeneration. Correspondingly, gelatin coating on PET film and fabric demonstrated excellent biocompatibility with HUVEC cells [54,55]. Gelatin-based biomaterials have been extensively developed for bone tissue engineering applications due to their biocompatibility with the organic component of bone extracellular matrix [56].

### Enhancing cell attachment with gelatin-coated hydrogels

**Fig 6:**
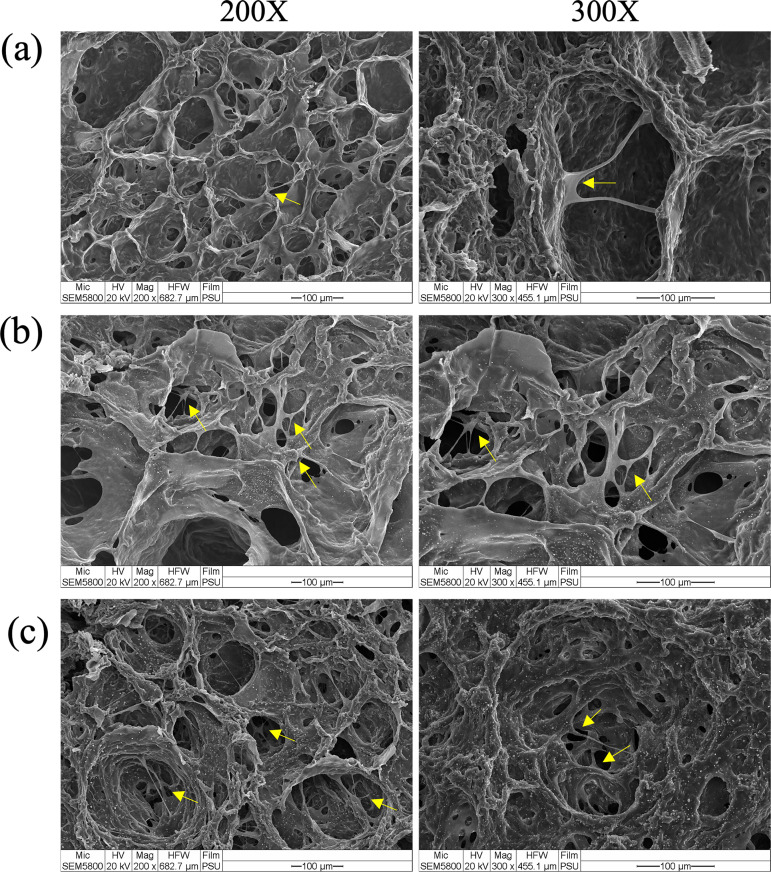
SEM images of HUVEC cell attachment on the hydrogels at magnifications of 200X and 300X. (a) uncoated hydrogel, (b) 0.1% gelatin-coated hydrogel, (c) 1% gelatin-coated hydrogel.

The initial cell adhesion to biomaterials is an essential process for biological responses, and plays a key role in promoting various physiological activities including cell proliferation, migration, differentiation, and extracellular matrix deposition [57]. The surface characteristics of the scaffold influence the processes of cell attachment and growth. Studies reported that modifications to the scaffold surface properties significantly impacted the initial attachment and subsequent growth of cells [58,59]. Several previous studies demonstrated that gelatin enhanced the attachment and proliferation behavior of endothelial cells, indicating its potential use in tissue engineering and regenerative medicinal applications [60,61]. HUVEC cells were cultured on the hydrogels for 7 days, and cell adhesion was observed using SEM (Fig 6). The results revealed that HUVEC cells were successfully cultured and attached on the hydrogel, with some spreading out. More cells were found on gelatin-coated hydrogels than on the uncoated hydrogel control, suggesting that gelatin coating improved cell attachment. The 0.1% and 1% gelatin-coated hydrogels exhibited favorable properties and provided a suitable environment for cell attachment as promising biomaterials in bone tissue engineering.

### Biological evaluation of gelatin-coated hydrogels containing VEGF-loaded sericin nanoparticles

**Fig 7:**
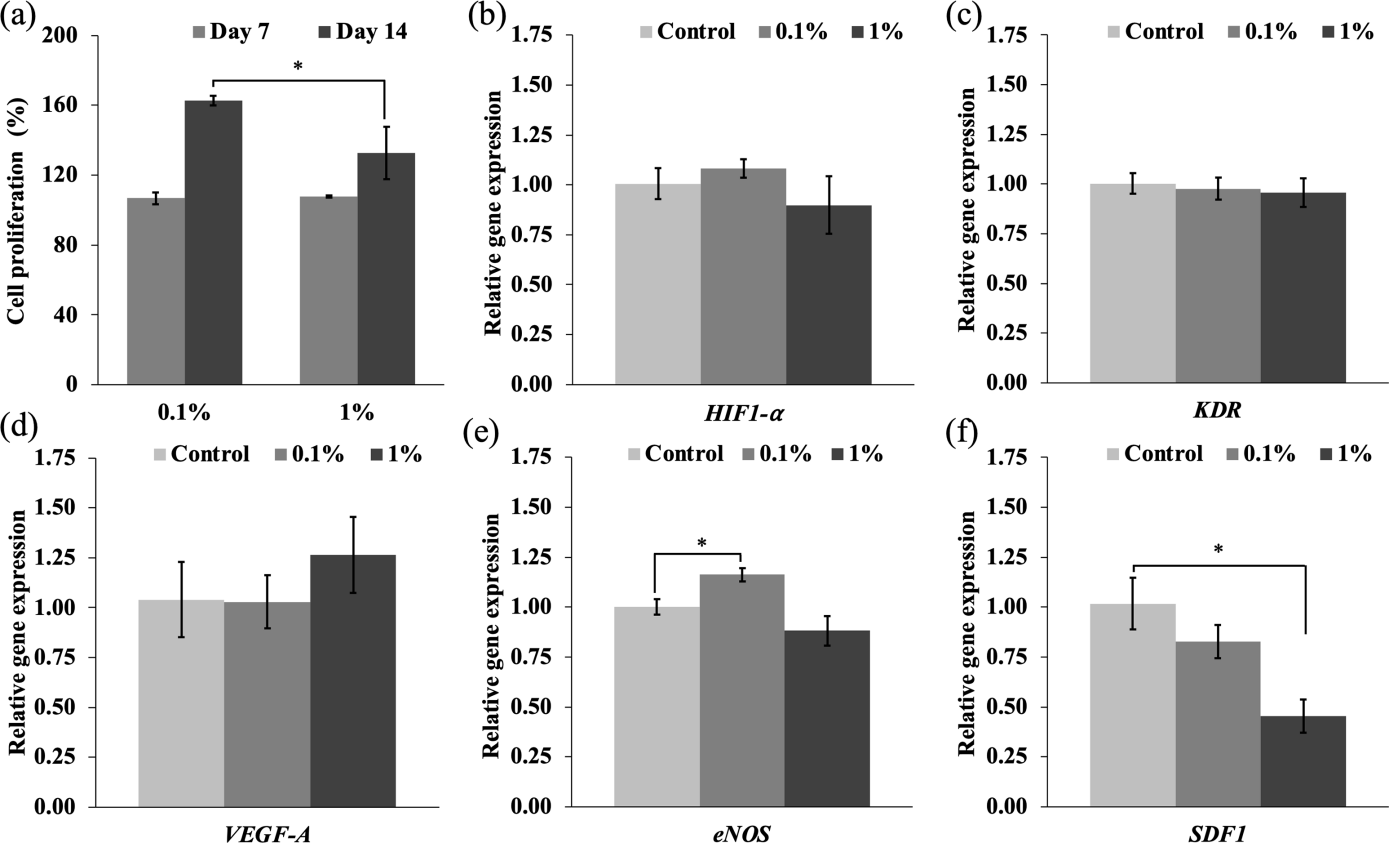
The effects of gelatin-coated hydrogel containing VEGF-loaded sericin nanoparticles on HUVEC cells. (a) HUVEC cell proliferation at 7 and 14 days, (b-f) the expression of angiogenic related genes (b) *HIF1-α*, (c) *KDR*, (d) *VEGF-A*, (e) *eNOS*, (f) *SDF1*. * represents a statistically significant difference (**p* *< 0.05).

Blood vessels facilitate the transportation of oxygen, nutrients, and waste products and are crucial for healing bone defects. Several studies developed scaffolds that enhanced the formation of blood vessels for tissue engineering applications [62,63]. Various approaches have been investigated to promote the regeneration and function of damaged tissues including developing a scaffold with bioactive materials, incorporating endothelial cells, and encapsulating growth factors within the scaffold [64–66]. These showed promising results in promoting tissue repair and regeneration. As mentioned above, VEGF plays a vital role in the proliferation of endothelial cells and the process of angiogenesis. Therefore, the stability of VEGF within a hydrogel scaffold is essential for the successful repair and regeneration of damaged tissues. In this study, VEGF was encapsulated in sericin nanoparticles and then incorporated into the hydrogel. The bioactivity of released VEGF on HUVEC cell proliferation was observed after 7 and 14 day treatment with VEGF release medium (Fig 7a). All the hydrogels were non-toxic and showed more than 100% cell viability. After 7 days of treatment with VEGF release medium, no significant difference in cell proliferation was found between the 0.1% and the 1% gelatin-coated hydrogels. After 14 days of treatment with VEGF release medium, cell proliferation of the 0.1% gelatin-coated hydrogel was significantly higher than the 1% gelatin-coated hydrogel, with an increase of over 160%. This result suggested that a higher concentration of hydrogel gelatin coating impacted the release of VEGF, with the high crosslink density on the hydrogel surface giving a lower proliferation rate. Sericin nanoparticles exhibited sustained release of VEGF for 14 days while maintaining bioactivity to promote the proliferation of HUVEC cells. Sericin nanoparticles and gelatin-coated hydrogel showed potential as an effective carrier for the sustained release of bioactive molecules.

VEGF is a major factor in angiogenesis to mediate the proliferation, migration, and differentiation of endothelial cells [67]. The expression levels of genes related to angiogenesis were quantitatively examined, as shown in Fig 7b–f. No significant differences in *HIF-1α* expression were observed between the groups. This gene is linked to tumor blood vessel growth triggered by hypoxia, being frequently overexpressed in cancer cells and a prevalent trait among many tumors [68,69]. This result implied that the hydrogels did not stimulate tumor angiogenesis. Likewise, the expression of *KDR* and *VEGF-A*, which can be influenced by hypoxia [70], did not show significant changes. The *eNOS* gene, which encodes the eNOS enzyme responsible for producing nitric oxide (NO) and serves as a key signaling molecule in angiogenesis [71], was upregulated by the hydrogels. The upregulation of *eNOS* by the 0.1% gelatin-coated hydrogel was statistically significant compared to the control and greater than the 1% gelatin-coated hydrogel. The VEGF level released from the 0.1% group was more compatible than from the 1% group to promote *eNOS* gene expression, stimulated through the phosphoinositide 3-kinase (PI3K)/protein kinase B (Akt)/eNOS signaling pathway [72]. The process is initiated by the binding of VEGF to its receptor (VEGFR) on endothelial cells, which triggers the PI3K/Akt/eNOS pathway. PI3K mediates Akt phosphorylation, which then promotes the phosphorylation of eNOS, enhancing NO production. Simultaneously, NO stimulates VEGF expression in endothelial cells [73]. The upregulation of the *eNOS* gene in the 0.1% gelatin-coated hydrogel occurred because increasing NO production promoted endothelial cell proliferation. The hydrogel downregulated the expression of the *SDF-1* gene and played multiple roles in orchestrating the complex process of blood vessel formation [74]. Results indicated that the release level of VEGF did not promote the expression of the *SDF-1* gene, suggesting that the hydrogels impacted the eNOS pathway and promoted angiogenesis.

### Antibacterial activities of gelatin-coated hydrogels containing VEGF-loaded sericin nanoparticles

**Fig 8:**
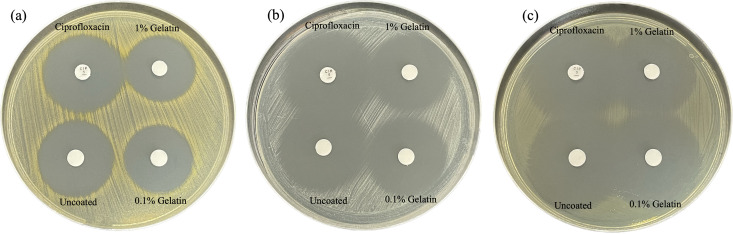
Inhibition zones of ciprofloxacin released from sericin/PVA hydrogels containing VEGF-loaded nanoparticles against bacteria. (a) *Staphylococcus aureus* ATCC 25923, (b) *Staphylococcus epidermidis* ATCC 12228, (c) *Escherichia coli* ATCC 25922.

Chronic osteomyelitis is a progressive inflammatory process caused by pathogens. Osteomyelitis management is challenging due to the resistance mechanisms developed by bacteria [75]. Antibiotic-loaded hydrogels, including ciprofloxacin, were synthesized and investigated as a local delivery system [18,76]. The disk diffusion method was performed to evaluate the antibacterial properties of ciprofloxacin released from the hydrogel against representative strains of Gram-positive bacteria (*S. aureus* ATCC 25923 and *S. epidermidis* ATCC 12228) and Gram-negative bacteria (*E. coli* ATCC 25922) (Fig 8 and Table 4). The inhibition zones of ciprofloxacin released from the uncoated, 0.1% gelatin, and 1% gelatin-coated hydrogels for all test isolates ranged from 29–37 mm, 26–36 mm, and 26–36 mm, respectively. The ciprofloxacin standard disk showed clear zones in the range of 30–37 mm, within the standard control range according to the CLSI breakpoints (22–30 mm for *S. aureus* ATCC 25923, ≥ 21 mm for all staphylococci including *S. epidermidis* ATCC 12228, and 29–38 mm for *E. coli* ATCC 25922) [77]. These results indicated that ciprofloxacin released from the hydrogel was active and effective against pathogens. Ciprofloxacin and tetracycline-loaded materials exhibited inhibition zones against *S. aureus* and *E. coli* [78]. The antibacterial activity of the ciprofloxacin-loaded hydrogel showed an inhibition zone against *E. coli* but zone diameters varied due to the formulation [79].

**Table 4:** Inhibition zone diameters of ciprofloxacin release from sericin/PVA hydrogels containing VEGF-loaded nanoparticles against bacteria.

Bacteria	Diameter of inhibition zone (mm)			
	Nanoparticle-loaded hydrogel			Control
	Uncoated hydrogel	0.1% gelatin	1% gelatin	Ciprofloxacin (5 µg)
*S. aureus* ATCC 25923	29 ± 0.00	26 ± 0.71	26 ± 0.00	30 ± 0.00
*S. epidermidis* ATCC 12228	31 ± 0.71	30 ± 0.71	29 ± 0.00	35 ± 0.00
*E. coli* ATCC 25922	37 ± 0.00	36 ± 0.00	36 ± 0.71	37 ± 0.00

The hydrogel showed effective antibacterial activities against bacteria, with gelatin concentrations affecting the amount of drug release. Gelatin was applied to coat and control the release of bioactive compounds [80]. A previous report found that paclitaxel release from liposomes depended on the concentration of gelatin coating [81]. These findings indicated that ciprofloxacin could be successfully loaded into the hydrogel and then released to function against bacteria. The sericin/PVA hydrogel maintained effective antibacterial activity against Gram-positive and Gram-negative bacteria [18]. However, additional research on clinically relevant isolates is required to validate our findings and offer robust evidence to support their use in therapeutic outcomes.

## Conclusions

Sericin/PVA hydrogels were successfully optimized to enhance angiogenesis by incorporating sericin nanoparticles as a VEGF carrier. Gelatin-coated hydrogels demonstrated suitable physical and mechanical characteristics with appropriate release rates and promoted the growth of cells, especially in HUVECs, while also exhibiting antibacterial properties. Sericin/PVA hydrogels show promise as a biomaterial implant to alleviate chronic osteomyelitis through the promotion of angiogenesis.
